# Halide Coordination Motifs in Deep Eutectic Solvent Building Blocks Characterized by Helium Nanodroplet Infrared Action Spectroscopy

**DOI:** 10.1021/acs.jpca.6c02885

**Published:** 2026-08-11

**Authors:** Miyuru M. Wellalage, Maleesha T. Fernando, Katja Ober, América Y. Torres-Boy, Madeline Schultz, Nwanne D. Banor, Emily M. Molino, Jenna E. Lees, Gert von Helden, Daniel A. Thomas

**Affiliations:** † Department of Chemistry, 4260 University of Rhode Island, Kingston, Rhode Island 02881, United States; ‡ Fritz Haber Institute of the Max Planck Society, 14195 Berlin, Germany

## Abstract

Deep eutectic solvents (DESs) are a broad class of multicomponent solvents that allow for tuning of properties through informed selection of constituent molecules. A comprehensive assessment of DES intermolecular interactions and their link to macroscopic attributes facilitates component selection. In this work, we employ infrared (IR) action spectroscopy in helium nanodroplets to characterize isolated halide–molecule clusters relevant to DESs. Complexes of urea, ethylene glycol, glycerol, and choline with chloride or bromide were generated by electrospray ionization, isolated in an ion trap, and captured in helium nanodroplets for spectroscopic characterization in the IR fingerprint region. For urea–chloride clusters, halide–molecule rather than molecule–molecule interactions dominate, resulting in a shallow potential energy surface with multiple orientations of the urea molecules differing primarily in their relative positioning. For clusters with strong competition between halide–molecule and intermolecular hydrogen bonding, as in glycerol–halide clusters, the potential energy surface becomes more complex, featuring multiple low-energy configurations with distinct hydrogen-bonding networks. Choline–chloride clusters exhibit possible two-point (OH + CH) and three-point (3CH) halide coordination motifs. Comparison to condensed-phase DES FT-IR spectra reveals distinctions between isolated clusters and bulk solution, highlighting the importance of assessing both intermolecular and halide-molecule interactions to accurately represent macroscopic DES structure.

## Graphical abstract

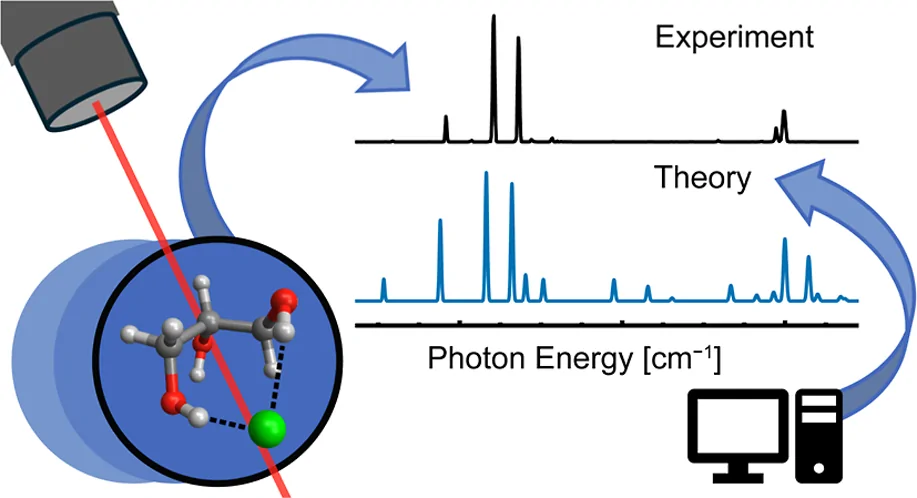

## Introduction

With increasing interest in “tunable” solvent properties and an ever-growing search for solvents with decreased negative health and environmental impacts, deep eutectic solvents (DESs) have naturally become a prominent research topic.^1–4^ Although the DES designation has been applied to a broad subset of systems, these solvents typically comprise distinct hydrogen-bond donor and acceptor molecules, with intermolecular interactions between constituents substantially decreasing the melting point at the optimal eutectic composition.^1^ Notably, many DESs can be prepared from low-toxicity, relatively inexpensive components, contributing to their broad applicability.^2,5–7^ In the most well-established DES formulations, the hydrogen bond acceptor is a quaternary ammonium salt, such as choline chloride, and the hydrogen bond donor is a polar organic molecule such as urea, ethylene glycol, or glycerol.^1,8,9^ Over time, the range of known DESs has expanded to include several subclasses, which may be categorized by their chemical composition (e.g., types I–IV)^1,9^ or by specific properties (e.g., hydrophobic DESs or natural DESs).^3,10^ Although these solvents are diverse, they are united by the concept of multimodal noncovalent solvent–solvent and solute–solvent interactions that can be tailored for specific applications, such as liquid–liquid extraction,^11–13^ electrochemical processing,^14,15^ or synthetic reaction control.^16–18^

Elucidation of the structural motifs underlying the properties and dynamics of DESs is central to developing solvents with attributes tailored for specific applications.^19–24^ Numerous experimental methods have been applied to assess the structural properties of DESs, including vibrational spectroscopy,^25–28^ nuclear magnetic resonance (NMR) spectroscopy,^29,30^ inelastic neutron scattering,^26^ broadband dielectric spectroscopy,^31,32^ and fluorescence spectroscopy.^16,33–35^ Among the most informative results have been those obtained by neutron diffraction experiments in combination with empirical potential structure refinement (EPSR), which together provide information on the nearest–neighbor interactions of DES constituents.^36–39^ Experimental methods have also been complemented by extensive theoretical efforts, including classical molecular dynamics,^27,40–43^ ab initio molecular dynamics,^38,40,43,44^ and cluster geometry optimization using electronic structure methods.^26,27,45–47^

Although general structural properties have been identified for the most common DES formulations, significant uncertainty remains regarding the prevalence and structural contributions of specific intermolecular interactions, most notably those between donors and charged moieties or among donors themselves (which may also act as hydrogen-bond acceptors).^1^ Complementary to condensed-phase analysis of solvent structure, infrared (IR) action spectroscopy of isolated ionic clusters^48–55^ can be applied to investigate structural motifs relevant to DESs. In this approach, ionic clusters are generated via electrospray ionization, isolated in vacuum using mass spectrometry, and characterized by IR action spectroscopy. Taking advantage of the high degree of control and specificity afforded by spectroscopic characterization under vacuum conditions, this technique enables confident assignment of cluster structure. By analyzing clusters varying in size and composition, structural motifs of clusters can then be compared to condensed-phase behavior in a “bottom-up” approach. Such methodology has previously been applied to charged water clusters to examine proton shuttling mechanisms in aqueous solution,^55–60^ the microsolvation of common charged analytes,^61–65^ and the investigation of long-range ordering of water molecules in ion-containing nanodroplets.^66–68^ Although differences between cluster and bulk structure exist,^69–71^ the spectroscopic interrogation of isolated clusters can still provide valuable structural insight.

In this work, we analyze the structure of elementary clusters comprising components of common DESs, focusing on halide complexes with urea, ethylene glycol, glycerol, and choline. We present IR action spectra obtained following capture of ionic clusters in helium nanodroplets, where thermalization to 0.4 K results in highly resolved spectra of low-energy structures.^72–75^ We assess the vibrational modes giving rise to spectral features in the fingerprint region by referencing normal modes computed from electronic structure calculations, providing insight into the ionic hydrogen bonding structure. We also compare spectra of isolated clusters to condensed-phase IR spectra to assess possible contributions of the observed structural motifs in solution.

## Experimental Section

### Materials

For DES preparation, choline chloride (98%), ethylene glycol (99%), urea (99%), and glycerol (99%) were purchased from Bean Town Chemical Corporation (Hudson, NH, USA). For cluster generation, HPLC-grade water, HPLC-grade acetonitrile, choline chloride (99%), ethylene glycol (>99%), glycerol (>99.5%), tetrabutylammonium chloride (>97%), and tetrabutylammonium bromide (>98%) were purchased from Merck (Darmstadt, Germany).

### DES Preparation and Characterization

The experimental setup for DES preparation is depicted in Figure S1. In a 50 ml round-bottom flask, choline chloride and a hydrogen bond donor were mixed in the ratios given in Table 1. The flask was kept in a water bath maintained within a specific temperature range (see Table 1) while continuously stirring the mixture under a nitrogen-gas atmosphere. After all the solids were dissolved, the temperature was reduced to room temperature, the water bath was removed, and the mixture was stirred for another 10–15 min. The prepared DES was stored in a precleaned, nitrogen-gas-flushed amber glass bottle covered with Al foil and stored in a desiccator. FT-IR spectra of DESs and DES components were acquired using a PerkinElmer Spectrum 100 attenuated total reflectance (ATR) spectrometer (PerkinElmer, Shelton, CT, USA).

**Table 1:** Description of the Prepared DESs

quaternary ammonium salt	hydrogen bond donor (HBD)	molar ratio between salt and HBD (salt: HBD)	preparation temperature range (°C)
choline chloride (ChCl)	urea	1:2	65–70
choline chloride (ChCl)	ethylene glycol (EG)	1:3	65–70
choline chloride (ChCl)	glycerol (Gly)	1:2	65–70

### Helium Nanodroplet IR Action Spectroscopy

Stock solutions of 500 μM tetrabutylammonium chloride (TBAC), 500 μM tetrabutylammonium bromide (TBAB), 1000 μM ethylene glycol (EG), and 500 μM glycerol (Gly) were prepared in acetonitrile. Stock solutions of 1000 μM urea and 1000 μM choline chloride (ChCl) were prepared in an acetonitrile–water (2:1 *v*/*v*) mixed-solvent system. Combinations of TBAC/EG (250 μM:500 μM), TBAB/EG (250 μM:500 μM), ChCl/urea (250 μM:250 μM) and ChCl/Gly (200 μM:400 μM) were electrosprayed to obtain ionic clusters of (EG + Cl^–^), (EG + Br^–^), (urea + Cl^–^), (2urea + Cl^–^), (Ch^+^ + 2Cl^–^), (Gly + Cl^–^) and (2Gly + Cl^–^), respectively.

The instrumental setup has been described in detail previously^72–74^ and comprises two orthogonal axes. The first axis features a nanoelectrospray ionization (nESI) source, quadrupole mass filter, quadrupolar deflector, and time-of-flight detector. The second axis contains a helium nanodroplet source, a variable-temperature hexapole ion trap, an IR irradiation region, and a second time-of-flight detector. Ions generated and selected within the first axis are transferred via the quadrupole deflector to the hexapole ion trap of the second axis, wherein they are held in an axial trapping potential of ca. 3 V and cooled to ca. 90 K by collisions with He buffer gas.^76^ Individual ions are then captured by a traversing beam of helium nanodroplets and thermalized within the droplet to ca. 0.4 K. The ion-doped droplets overcome the trapping potential and proceed to the irradiation region, where they are irradiated with IR photons generated by the free-electron laser (FEL) facility of the Fritz Haber Institute.^77^ Resonant photon absorption yields evaporation of helium, resulting in the formation of bare ions.^75,78^ These ions are directed to a time-of-flight detector, and monitoring of ion signal as a function of photon energy yields an IR action spectrum.

Experiments were performed with the pulsed helium nanodroplet source operating at a backing pressure of ca. 69 bar and a temperature of 23 K, yielding nanodroplets with an average size of ca. 24,000 He atoms.^73^ Data points were collected between 800 and 1750 cm^–1^ in 1 cm^–1^ or 2 cm^–1^ steps, and the ion trap was refilled between each measurement step. At each stepped wavelength, the spectrum intensity was obtained by integrating the intensity of the target *m*/*z* ion signal from the average of 25 helium nanodroplet beam pulses synchronized with FEL macropulses. A factor of (laser power × wavenumber)^−1^ was applied to intensities to adjust for variations in photon fluence. To achieve operation in a pseudolinear response regime,^75^ laser power was adjusted to avoid line saturation or low intensity in different spectral regions. Each region was scanned at least twice at a given laser power and averaged to ensure reproducibility. After acquisition, spectra were scaled by the intensity of lines overlapping between scans to generate a composite spectrum.

### Electronic Structure Methods

Conformational sampling of ionic DES clusters was carried out using the conformer-rotamer ensemble sampling tool (CREST) of Pracht, Grimme et al.^79^ with the semiempirical tight binding-based quantum chemistry method (GFN2–xTB) with noncovalent interactions (nci) mode enabled.^80–83^ All the structures obtained by CREST within 25 kJ mol^–1^ of the lowest-energy structure were further optimized within the ORCA computational chemistry package^84,85^ using the hybrid density functional B3LYP^86–89^ with the empirical dispersion correction of Grimme and co-workers with Becke-Johnson damping (D3BJ)^90,91^ and the ma-def2-TZVP basis set^92–94^ and aug-cc-pVTZ/JK auxiliary basis set.^95–97^ For conformers of each system within 5 kJ mol^–1^ of the lowest-energy structure, optimization and frequency calculations were performed at the B3LYP, CAM-B3LYP,^98^ and RI-MP2 ^99,100^ levels of theory with the same basis set and auxiliary basis set. Frequencies were scaled by 0.985, 0.965, and 0.97 for B3LYP, CAM-B3LYP, and RI-MP2, respectively, with scaling factors based on empirical fitting to experimental spectra. Anharmonic IR frequencies were obtained by the VPT2 method at the B3LYP­(D3BJ)/ma-def2-TZVP level of theory with the aug-cc-pVTZ/JK auxiliary basis set following geometry optimization with very tight constraints (ORCA keyword VERYTIGHTOPT).^85,101–103^ Harmonic intensities of fundamental modes were combined with anharmonic intensities for overtones and combination bands. Computed IR spectra were constructed by convolution of computed IR lines and a Gaussian function with a width of 0.3% (full width at half-maximum) of the line frequency. Final relative energies were assessed with the inclusion of a harmonic zero-point energy (ZPE) correction, Δ­(*E* + ZPE).

## Results & Discussion

### IR Action Spectroscopy of Clusters

This study focuses on the interactions of halides with other DES components, which are central to DES properties in the condensed phase.^36–39^ To explore the fundamental attributes of these interactions, halide–molecule clusters were generated by nESI and probed by IR action spectroscopy in He nanodroplets. We first examine the complex between the simple diol ethylene glycol (EG) and Cl^–^ or Br^–^, the IR spectra of which are shown in Figure 1a,c, respectively. The spectra are dominated by lines at ca. 1080 and 1110 cm^–1^ (lines a_1_, a_2_, b_1_, b_2_), with the lines of (EG + Br^–^) slightly red-shifted from those of (EG + Cl^–^) (Table S1). Only a single low-energy structure featuring two ionic hydrogen bonds between EG and the halide was found from conformational sampling for these systems. IR spectra computed at the B3LYP/ma-def2-TZVP level of theory (0.985 scaling factor) are depicted in Figure 1b,d and show strong agreement with the experimental spectra for the most intense spectral lines. The spectra computed with the B3LYP functional are presented throughout this work, as this method was found to give better agreement with experimental spectra than CAM-B3LYP or RI-MP2 (see Figures S2 and S3). The two most intense lines in each spectrum are assigned to in-phase and out-of-phase stretching of the two C–O bonds. Agreement of the experimental and theoretical spectra is poorer for the low-intensity lines in the region of CH and OH bending modes, with no clear theoretical match to lines a_3_ and b_3_ and a poorer match for the diffuse lines a_5_ and b_5_. Anharmonic IR spectra computed with the VPT2 method did not yield a better fit for these low-intensity lines (Figure S5), although this approach can be highly sensitive to electronic structure method and large amplitude motions.^103^ Therefore, anharmonic features such as combinations bands and overtones may still account for some discrepancy between the experimental and predicted IR spectra. Comparing the spectra of Cl^–^ and Br^–^ complexes, the change in halide has little effect on the spectra in the fingerprint region, consistent with the minimal change in geometry between the two adducts, and chloride-bound complexes were consequently chosen as the focus of this work. Larger clusters of EG with halides were not readily generated using the instrumentation available.

**Figure 1:**
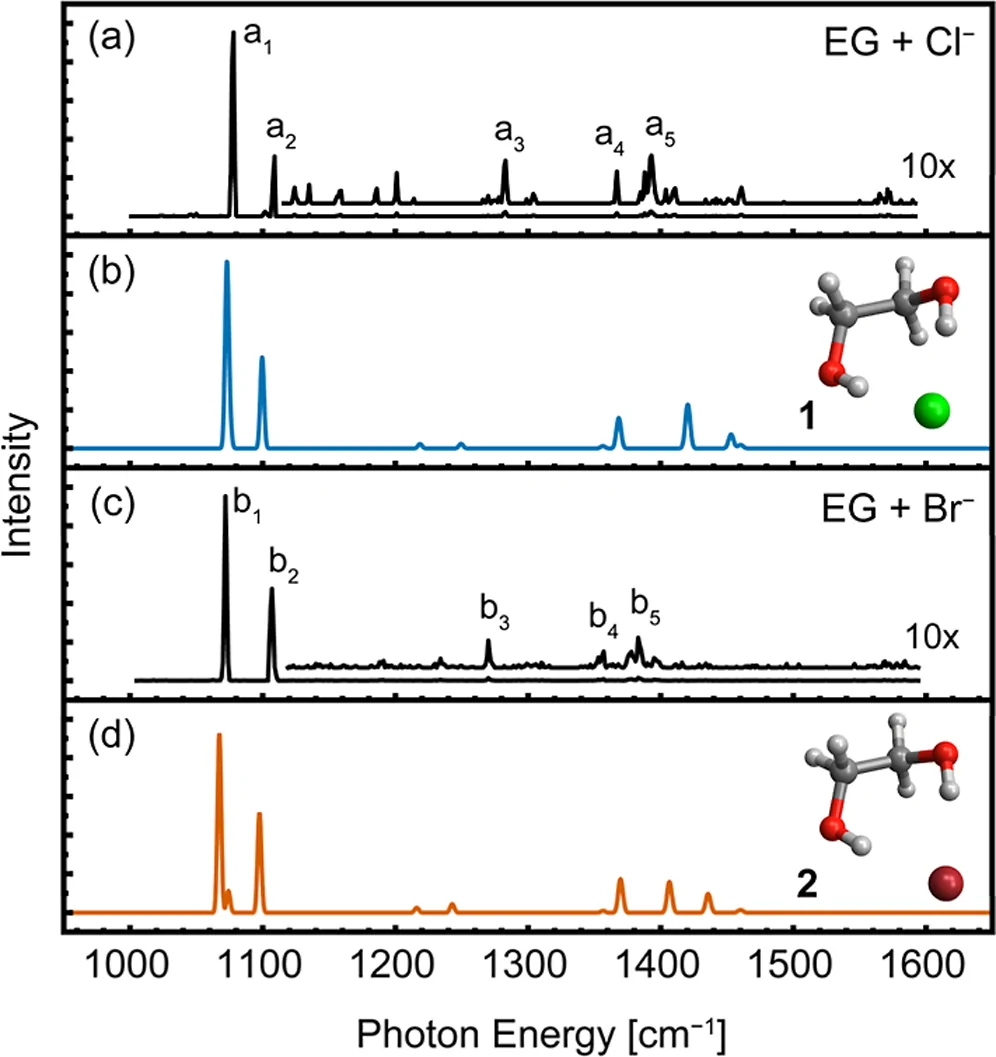
He nanodroplet IR action spectra of the complex between ethylene glycol and Cl^–^ (a) or Br^–^ (c). Computed IR spectra for the identified low-energy structures are shown in (b,d) (B3LYP/ma-def2-TZVP, 0.985 scaling factor).

Moving from ethylene glycol to glycerol (Gly), the conformational complexity increases substantially owing to the numerous possible hydrogen bonding configurations. The IR action spectra for the complex between one or two glycerol molecules and chloride are shown in Figure 2a,c, respectively. As with EG, the most intense lines are found in the region of C–O stretching modes (c_3_, c_4_, d_2_–d_4_), with moderate-intensity lines also observed in the region of CH and OH bending modes (c_5_, c_6_, d_6_) and in the region of skeletal stretching modes (c_1_, c_2_, d_1_). For the complex (Gly + Cl^–^), a clear global-minimum-energy structure **3** was identified (Scheme 1) that features hydrogen bonding between Cl^–^ and the two terminal hydroxyl groups, with the central hydroxyl group acting as an intramolecular hydrogen-bond donor.^104^ The predicted IR spectrum for this structure (Figure 2b) shows good agreement with experiment, confirming the vibrational mode assignments. Note that the larger-than-predicted intensity difference between weak and strong lines in the experimental spectrum can arise from the nonlinear response of the He nanodroplet action spectroscopy approach, in which the detected ion signal can exhibit a sigmoidal dependence on single-photon absorption probability.^75^

**Figure 2:**
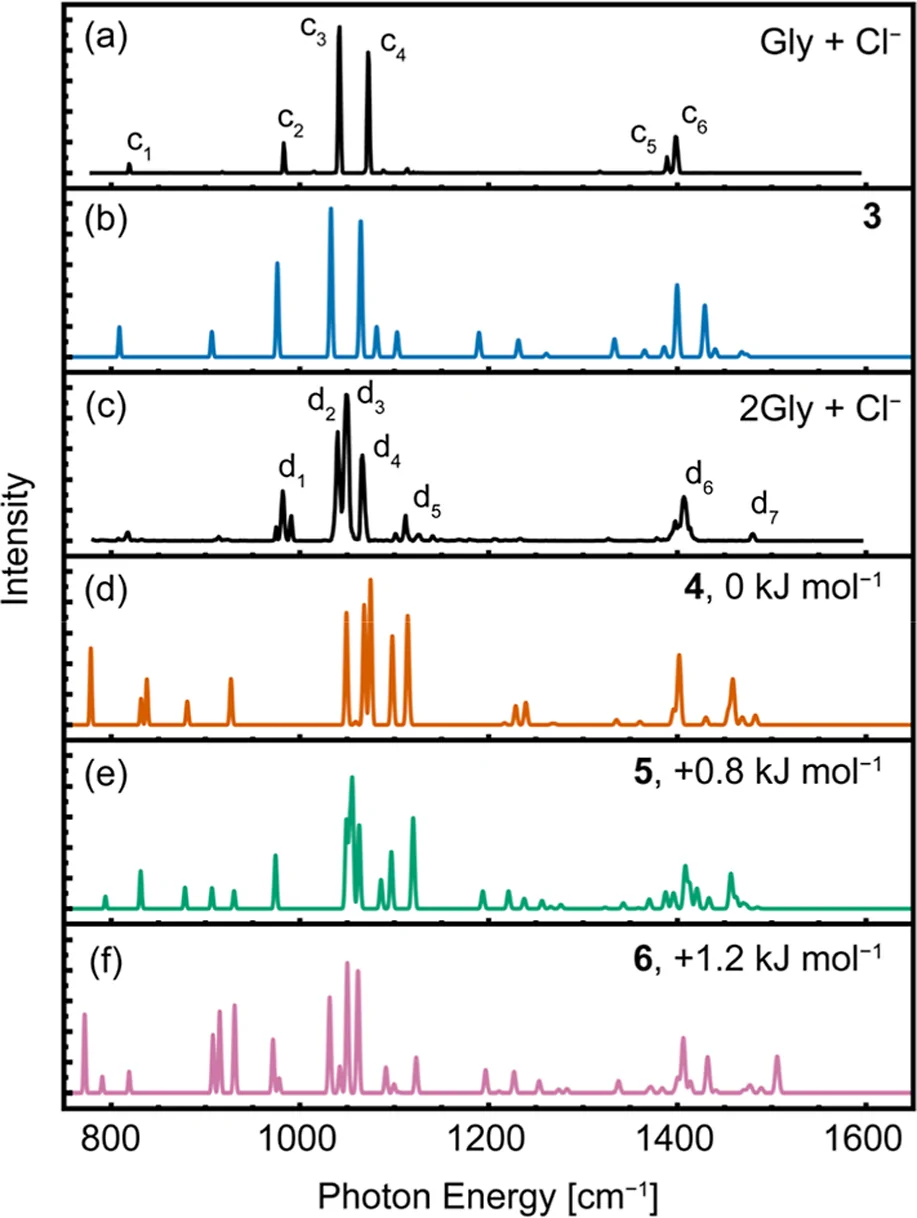
He nanodroplet IR action spectra of the complex between Cl^–^ and one (a) or two (c) glycerol molecules. Computed IR spectra (B3LYP­(D3BJ)/ma-def2-TZVP, 0.985 scaling factor) for the identified low-energy structures are shown in (b,d–f), with the corresponding structures shown in Scheme 1.

**Scheme 1:**
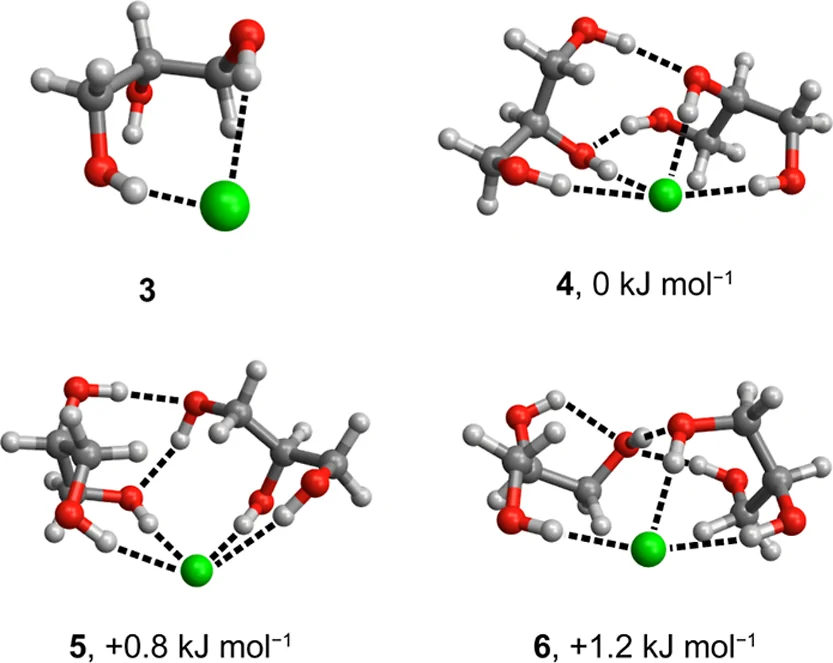
Low-Energy Structures of (Gly + Cl^–^) (**3**) and (2Gly + Cl^–^) (**4–6**)

For (2Gly + Cl^–^), the potential energy surface is significantly more complex, with 11 conformers identified within 5 kJ mol^–1^ of the global-minimum structure. The three lowest-energy conformers are shown in Scheme 1 (structures **4**–**6**), with the corresponding IR spectra shown in Figure 2d–f. Additional structures and spectra are shown in Figure S4. Each of the low-energy structures features a unique hydrogen-bonding configuration, with both structures **4** and **5** having four OH···Cl^–^ hydrogen bonds and structure **6** having three OH···Cl^–^ hydrogen bonds. Comparing the experimental spectrum of (2Gly + Cl^–^) to the computed spectra of structures **4**–**6**, a clear match to a single configuration is not observed. The overall match of structure **4** is good, but the intensity of line d_5_ is not replicated, and no lines corresponding to d_1_ are predicted. The spectrum of structure **5** shows a slightly better match to experiment, with a predicted line in the region of line d_1_ but still an overprediction of the intensity of d_5_. The spectrum of structure **6** best replicates the experimental lines but also predicts several intense features near 900 cm^–1^ that are not observed experimentally. The addition of anharmonic corrections to the computed IR spectra does not substantially improve agreement with experiment, with small shifts for most lines and the addition of several high-intensity lines not observed experimentally (Figure S6). The experimental spectrum likely contains contributions from multiple low-energy conformers, as structures may be kinetically trapped during buffer-gas cooling to 90 K and while cooling to 0.4 K within the He nanodroplet.^105–108^ This possibility may explain the multiple maxima of line d_1_ and the broadness of lines d_2_–d_4_ and d_6_, which exhibit a full-width at half-maximum (fwhm) of ca. 5 cm^–1^ for (2Gly + Cl^–^) in comparison to ca. 3 cm^–1^ for analogous lines of (Gly + Cl^–^) (Table S8 and Figure S9).

Transitioning from hydroxyl to amino hydrogen-bond donors, the IR action spectra of one or two urea molecules complexed with Cl^–^ are shown in Figure 3a,c, respectively. The spectra of both the chloride-bound monomer and dimer are dominated by a line in the carbonyl stretching region (e_5_, f_3_), with lower-intensity lines in the NH bending (e_3_, f_2_) and CN stretching (e_1_, f_1_) ranges. Similar to (EG + Cl^–^), only a single low-energy configuration was observed for (urea + Cl^–^), as shown in structure **7** of Scheme 2. The computed IR spectrum of structure **7** (Figure 3b) replicates the most intense experimental lines and confirms their assignment as C=O stretching (e_5_), NH_2_ scissoring (e_3_), and CN asymmetric stretching (e_1_) modes. For (2urea + Cl^–^), the low-energy configuration was found to exhibit two-point coordination of both urea molecules to Cl^–^, with three nearly isoenergetic structures exhibiting this motif and differing only in the relative orientation of the two urea molecules (**8**–**10**, Scheme 2). A higher-energy structure in which one urea molecule is hydrogen-bonded to both Cl^–^ and the second urea (**11**, Scheme 2) was also found at the B3LYP and CAM-B3LYP levels of theory. As shown in Figure 3d–g, the IR spectra of structures **8**–**10** are largely indistinguishable, owing to the minimal perturbation arising from urea–urea interactions, and are largely consistent with the experimental spectrum of (2urea + Cl^–^) (Figure 3c). Anharmonic corrections show larger differences between the spectra of the three structures but do not broadly improve agreement with experiment (Figure S7). The broadening of the spectral lines of (2urea + Cl^–^) vs those of (urea + Cl^–^) (ca. 6 cm^–1^ vs ca. 4 cm^–1^ fwhm, Table S8) may reflect the population of multiple structures with slight differences in line positions.

**Figure 3:**
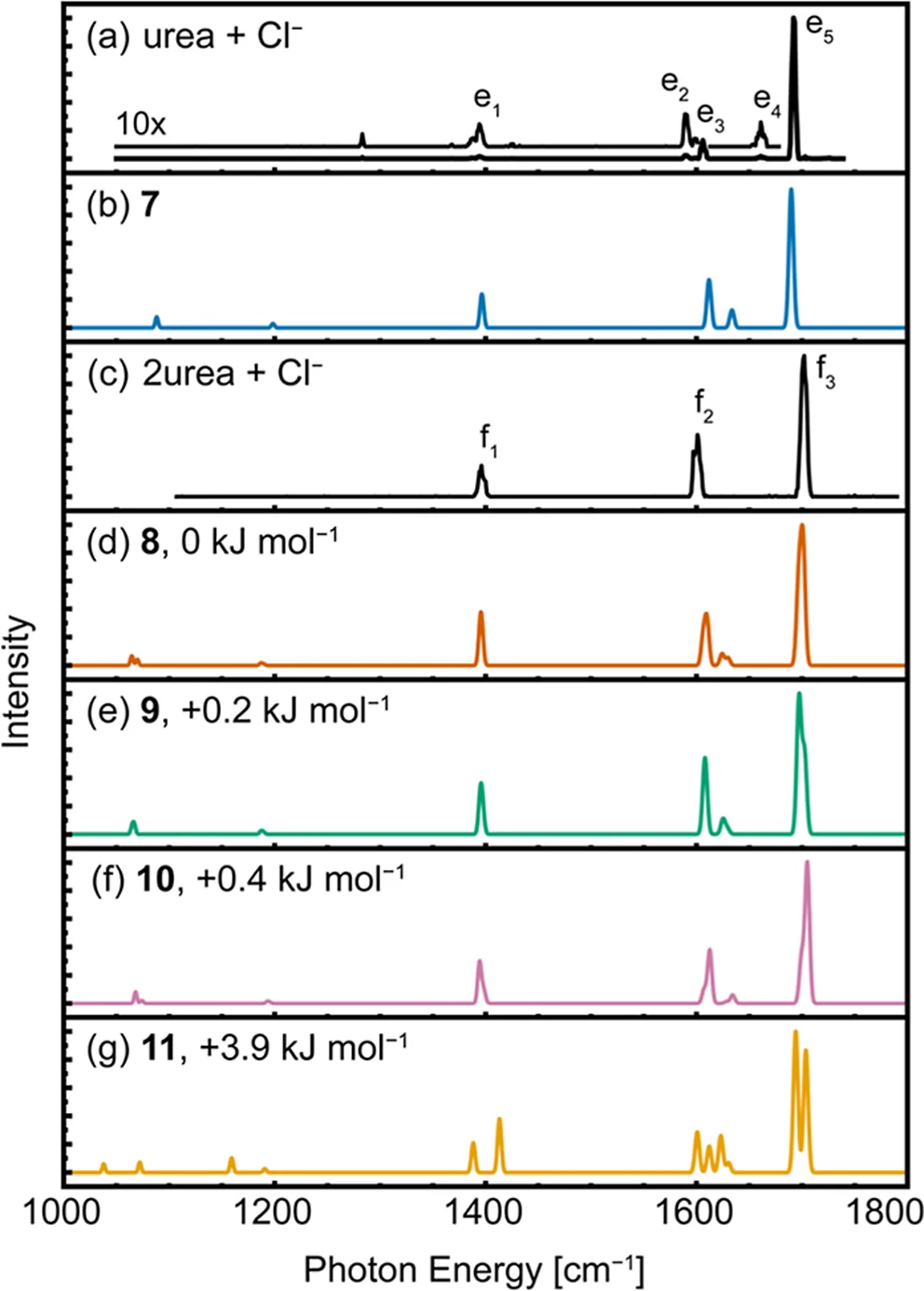
He nanodroplet IR action spectra of the complex between Cl^–^ and one (a) or two (c) urea molecules. Computed IR spectra for the identified low-energy structures are shown in (b,d–h), with the corresponding structures shown in Scheme 2 (B3LYP/ma-def2-TZVP, 0.985 scaling factor).

**Scheme 2:**
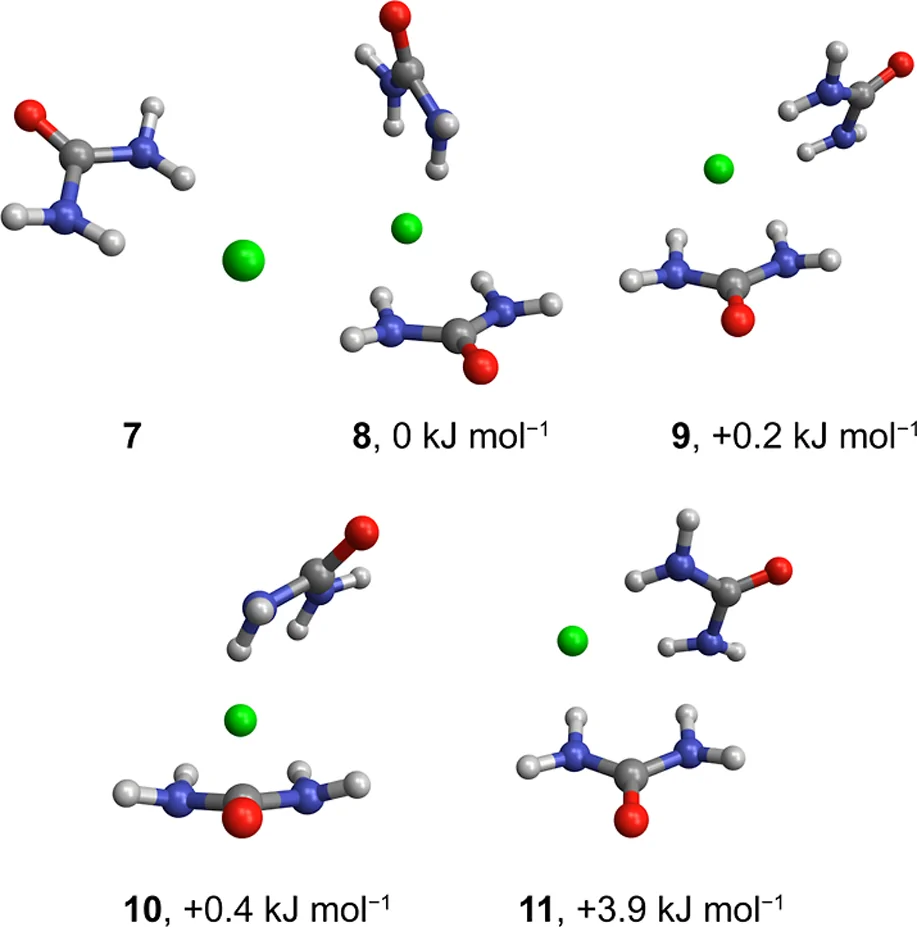
Low-Energy Structures of (Urea + Cl^–^) (**7**) and (2Urea + Cl^–^) (**8**–**12**)

Finally, we investigated the complex between choline (Ch^+^) and two chloride ions, (Ch^+^ + 2Cl^–^). As shown in Figure 4a, the experimental IR spectrum is characterized by several intense, sharp spectral lines, with the strongest line observed at 1101 cm^–1^ (g_4_). Conformational sampling and optimization of candidate structures identified three low-energy conformers that differ in the location of the two chloride ions around choline (Scheme 3). In the two lowest-energy structures **12** and **13**, one Cl^–^ is coordinated with an OH and CH group, and the second Cl^–^ is coordinated with three CH groups. In the higher-energy structure **14**, one Cl^–^ is coordinated with the OH and CH groups found on the same alkyl chain, and the second Cl^–^ is coordinated with two CH groups on two of the methyl substituents. The CH···Cl^–^ interactions in these complexes are likely particularly strong because of the substantial polarization of CH bonds in tetraalkylammonium ions.^109,110^ Referencing the computed vibrational modes, the experimental lines g_1_–g_4_ are assigned to OH bending (g_1_), tetraalkylammonium CN stretching (g_2_, g_3_), and CO stretching modes (g_4_). Lines g_5_–g_8_ are assigned as CH bending modes. Most experimental lines are reproduced in the computed IR spectrum of structure **12**, with the notable exception of line g_1_, which suggests a minor population of structures **13** and/or **14**, an assignment supported by the weak lines adjacent to g_2_ and g_4_ that may likewise arise from these structures. Major differences between the computed harmonic and anharmonic IR spectra are not observed for the low-energy structures of this complex (Figure S8). Note that the complex between choline (Ch^+^) and a single chloride ion, (Ch^+^ + Cl^–^), cannot be examined using this technique because the overall charge of the cluster is neutral, preventing the isolation and trapping required for spectroscopic analysis.

**Figure 4:**
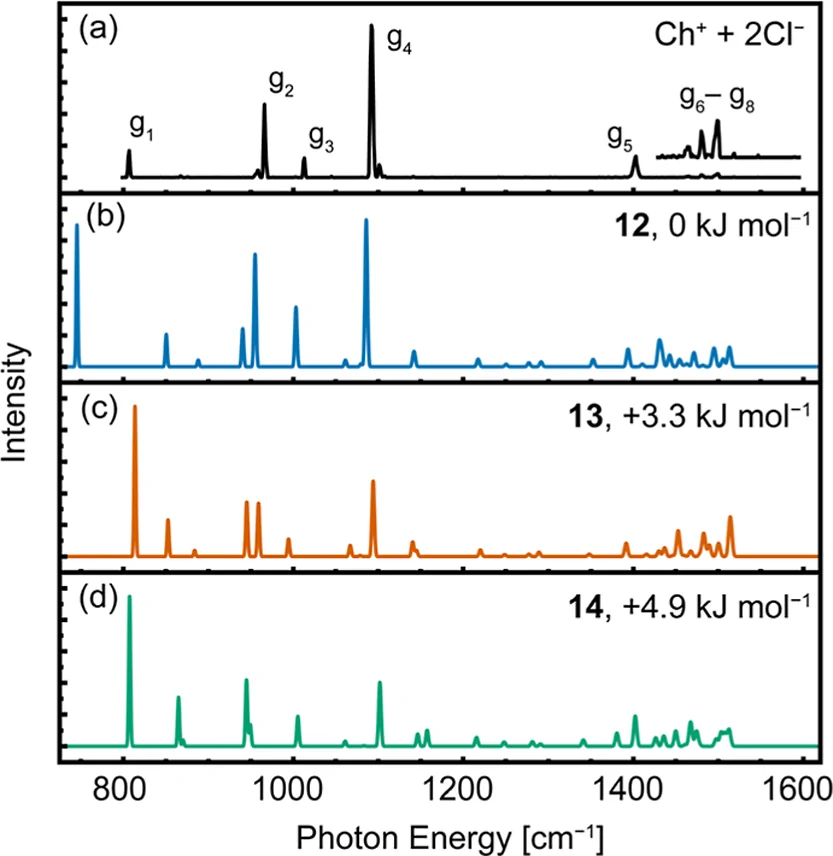
He nanodroplet IR action spectrum of the complex between choline (Ch^+^) and two Cl^–^ ions (a). Computed IR spectra for the identified low-energy structures are shown in (b–d), with the corresponding structures shown in Scheme 3 (B3LYP/ma-def2-TZVP, 0.985 scaling factor).

**Scheme 3:**
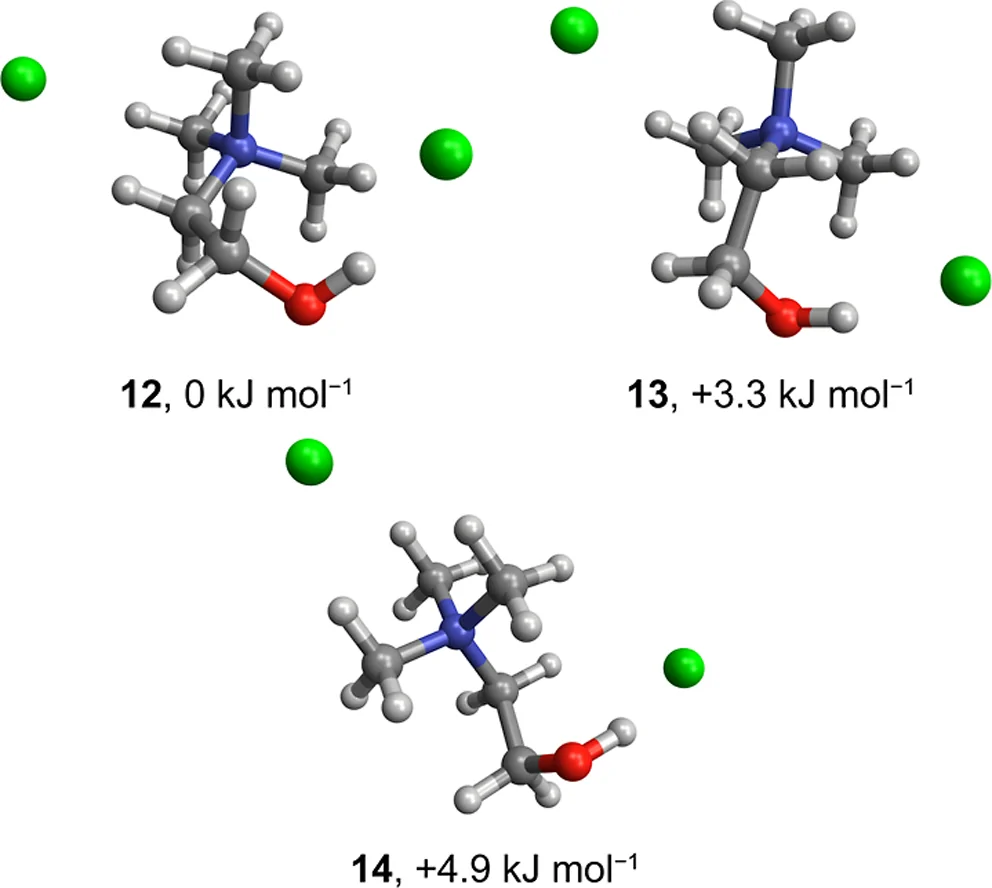
Low-Energy Structures of (Ch^+^ + 2Cl^–^)

### Comparison of Condensed-Phase and Cluster IR Spectra

To assess potential relevance of cluster structure to condensed-phase properties, it is useful to compare cluster and condensed-phase IR spectra. Shown in Figure 5 are the FT-IR spectra of choline chloride (ChCl, Figure 5a), urea (Figure 5b), and the 1:2 ChCl/urea DES (reline, Figure 5c). Figure 5d–f show the IR action spectra of (urea + Cl^–^), (2urea + Cl^–^), and (Ch^+^ + 2Cl^–^), respectively. In the urea–chloride clusters, the spectral lines assigned to carbonyl stretching modes (e_5_ and f_3_) are blue-shifted from the corresponding condensed-phase band at 1660 cm^–1^.^26^ This result is attributed to the absence of hydrogen-bond donors to the carbonyl moieties in the clusters, which in solution result in a red-shift of the carbonyl stretching modes. The NH bending modes (e_3_ and f_2_) show only a minimal shift in comparison to the condensed-phase bands of the DES (1606 cm^–1^). The relative positions of these lines indicate a nonplanar urea structure in both the DES and the isolated clusters.^26^ In contrast, the crystalline urea spectrum shows the opposite ordering of carbonyl stretching and NH bending modes, consistent with a planar urea structure stabilized by uniform hydrogen bonding to the carbonyl.^111^ The asymmetric CN stretching modes (e_1_, f_1_) are substantially red-shifted from the corresponding band of the bulk DES (1430 cm^–1^),^26^ which is itself red-shifted from the crystalline urea band. However, the CN asymmetric stretching line positions of the cluster are comparable to those of gas-phase urea,^112^ indicating a similarly pyramidal structure of urea in the chloride-bound complexes. This result again reflects the absence of hydrogen bond donors to the carbonyl groups in the isolated clusters; in condensed phases, hydrogen bonding to the carbonyl withdraws electron density from the carbonyl, enhancing nitrogen lone-pair delocalization and favoring a shift toward a more planar urea geometry.

**Figure 5:**
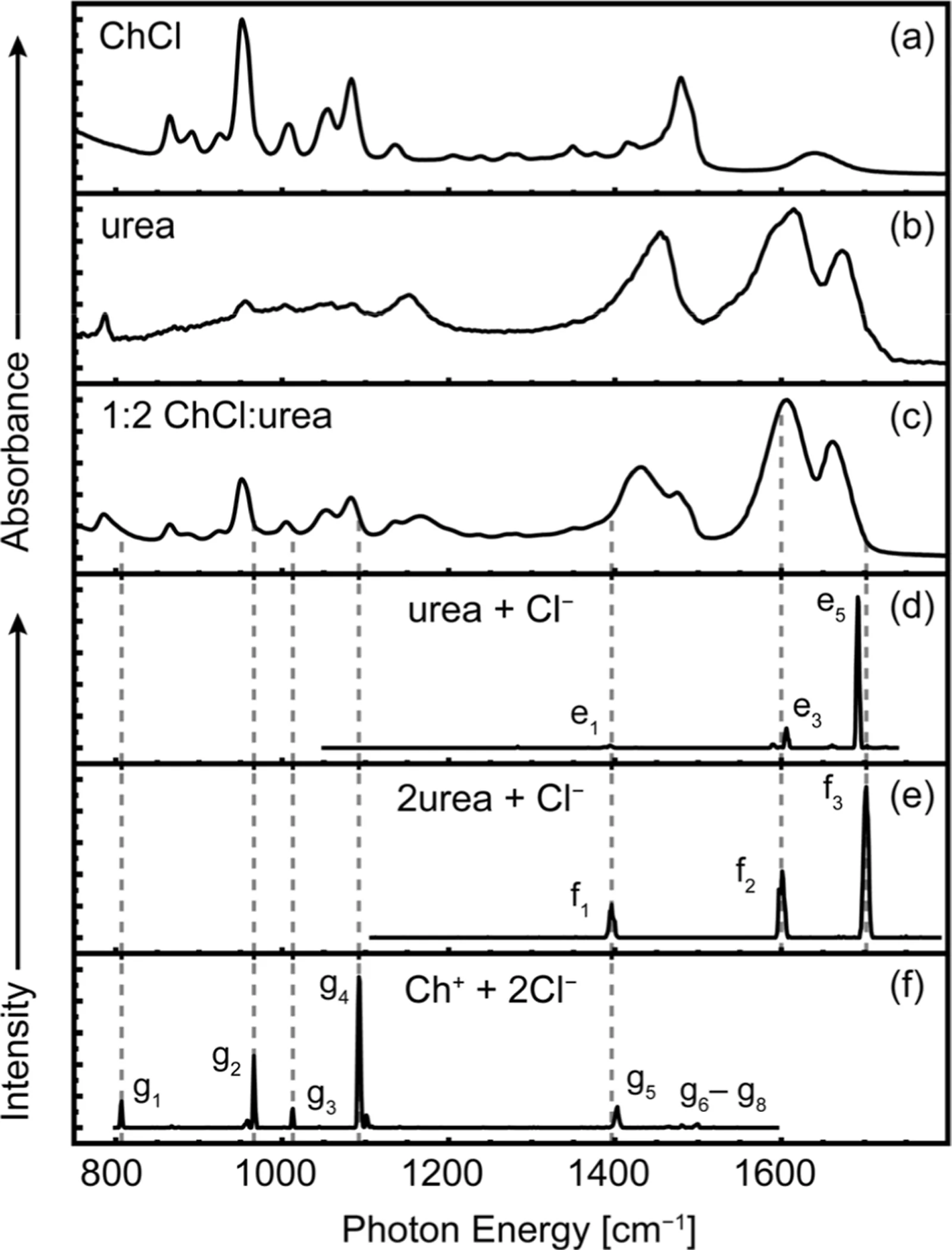
Comparison of condensed-phase FT-IR spectra of choline chloride (ChCl, (a)), urea (b), and 1:2 ChCl/urea DES (c) with IR action spectra of (urea + Cl^–^) (d), (2urea + Cl^–^) (e), and (Ch^+^ + 2Cl^–^) (f).

Turning to the cluster of choline with two chloride ions (Ch^+^ + 2Cl^–^), the line positions observed for CN and CCO stretching modes (g_2_–g_4_) are broadly similar to the analogous bands found in crystalline choline chloride as well as in the DES (952, 1005, and 1083 cm^–1^, respectively).^113,114^ The comparison of the cluster CH bending modes (g_5_–g_8_) to the corresponding lines in the DES or crystal is more challenging, as some bands are weak in this region and obscured in the reline IR spectrum by the dominant CN stretching band of urea (1430 cm^–1^).^26,114,115^ A general trend observed is that the low-energy CH-bending modes are more intense in the cluster (g_5_), whereas the higher-energy CH bending modes are more intense in reline (1476 cm^–1^). This result likely reflects the isolated interactions probed in the cluster (large transition dipole moment for select symmetric modes) vs the myriad intermolecular interactions sampled in the condensed phase (large transition dipole moment for asymmetric modes). The red-shifted OH bending mode g_1_ is not clearly observed in condensed-phase samples, although similar coordination of OH to Cl^–^ is expected both in crystalline ChCl and the DES.^26^ Interestingly, the three-point CH···Cl^–^ interaction identified in cluster structures **12** and **13** was recently suggested to occur prominently at the liquid/vapor interface of reline.^116^

To compare ethylene glycol and glycerol clusters to their condensed-phase counterparts, shown in Figure 6 are the FT-IR spectra of choline chloride (ChCl, Figure 6a), ethylene glycol (EG, Figure 6b), the 1:3 ChCl/EG DES (Figure 6c), glycerol (Gly, Figure 6e), and the 1:2 ChCl/Gly DES (glyceline, Figure 6f). Cluster IR action spectra of (EG + Cl^–^), (Gly + Cl^–^), and (2Gly + Cl^–^) are shown in Figures 6d,g,h, respectively. For (EG + Cl^–^), the two C–O stretching modes (a_1_ and a_2_) are blue-shifted from the corresponding condensed-phase bands (1035 and 1084 cm^–1^)^117,118^ and the reported line positions for gas-phase ethylene glycol (1055 and 1085 cm^–1^).^119^ This shift reflects the increased strength of the C–O bond upon ionic hydrogen bond formation. Notably, comparing the condensed-phase IR spectrum of pure EG (Figure 6b) to that of the DES (Figure 6c), such a blue-shift is not observed, suggesting weaker or less prominent EG···Cl^–^ interactions in the DES than in the clusters.

**Figure 6:**
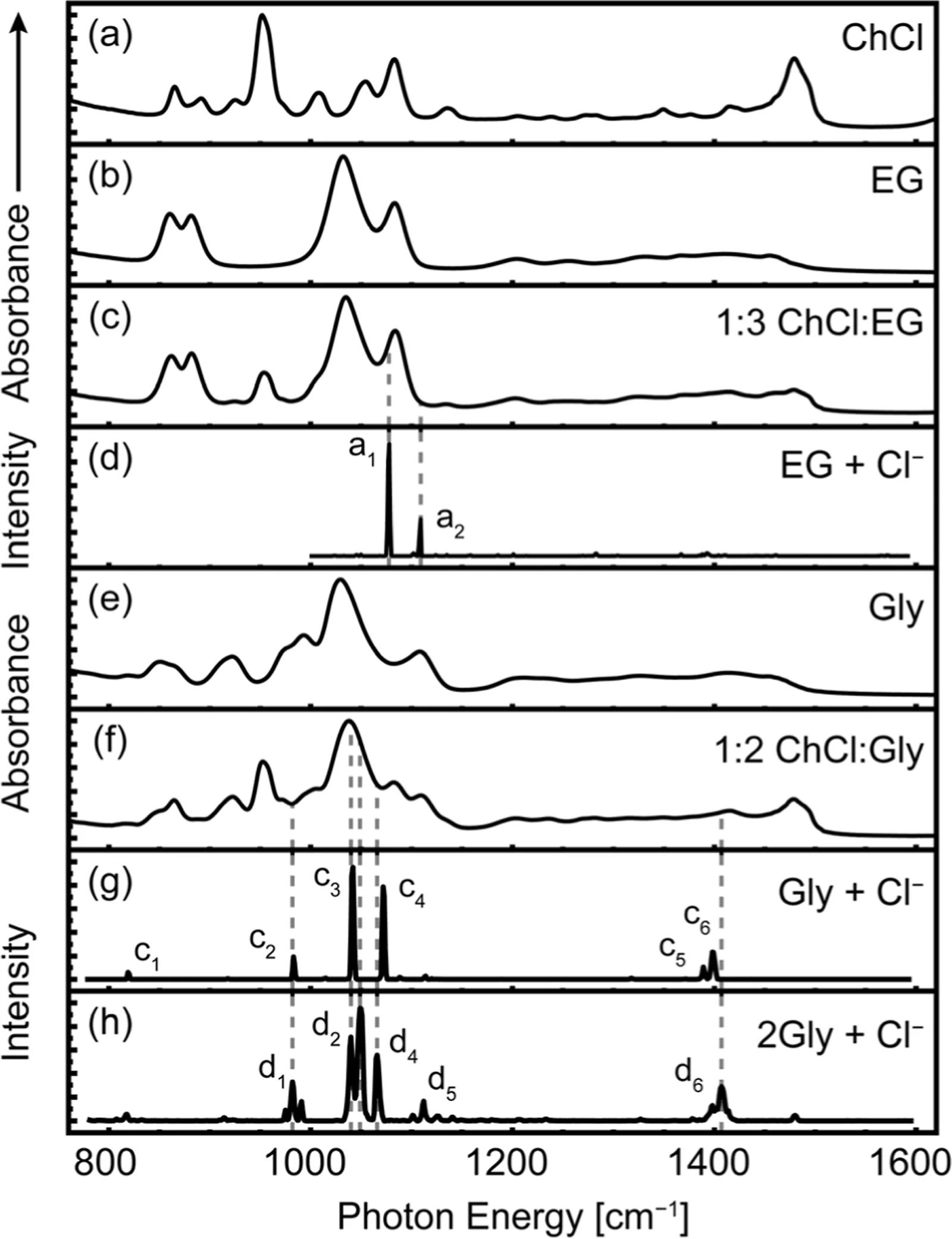
Comparison of condensed-phase FT-IR spectra of choline chloride (ChCl, (a)), ethylene glycol (EG, (b)), 1:3 ChCl/EG DES (c), glycerol (Gly, (e)), and 1:2 ChCl/Gly DES (f) with IR action spectra of (EG + Cl^–^) (d), (Gly + Cl^–^) (g), and (2Gly + Cl^–^) (h).

Turning to the glycerol-chloride clusters (Figure 6g,h), we observe that the line positions of C–O stretching modes (c_3_, c_4_, d_2_–d_4_) are closely aligned with the corresponding condensed-phase band of neat glycerol and the 1:2 ChCl/Gly DES (1034 cm^–1^),^120–123^ with the line positions and breadth more closely reflecting the bulk DES in the larger cluster size. The agreement in the position of the coupled CH and OH bending modes (c_5_, c_6_, d_6_) likewise improves with increasing cluster size, showing a broadening and blue shift toward the DES maximum of 1415 cm^–1^.^120,124,125^ The lines associated with skeletal stretching modes (c_2_, d_1_) also become more complex in the larger cluster, but the corresponding condensed-phase bands are not well-established from previous studies. Overall, the improved correspondence is attributed to the numerous intermolecular hydrogen bonding interactions in (2Gly + Cl^–^) clusters, which better reflect the complexity of hydrogen-bonding configurations in the condensed phase. Finally, we note that the choline features identified above in the reline spectrum may also contribute to the EG and Gly-based DES spectra, though they overlap with C–O stretching and CH/OH bending bands of ethylene glycol and glycerol.

## Conclusion

We have applied helium nanodroplet IR action spectroscopy to examine the structure of isolated halide-molecule clusters relevant to deep eutectic solvent composition. For clusters dominated by halide–molecule interactions, such as (2urea + Cl^–^), a shallow potential energy surface is observed that reflects minimal steric constraints on the positioning of molecules around the halide center. When additional strong intermolecular interactions are introduced, as in (2Gly + Cl^–^), the potential energy surface becomes significantly more complex, exhibiting numerous local minima with distinct hydrogen-bonding configurations. Clusters of (Ch^+^ + 2Cl^–^) show two-point OH and CH interactions as well as three-point CH interactions as possible coordination motifs between choline and chloride ions. Comparing the cluster spectra to those of condensed-phase DESs, we find that correspondence is best when both halide–molecule and molecule–molecule interactions are represented in the interrogated cluster. These results may provide useful benchmarks for validating computational approaches for the study of DES structure. Moving forward, this work would be complemented by the examination of larger clusters relevant to DES composition to better capture intermolecular interaction motifs. In addition, acquisition of IR spectra in the hydrogen-stretching region would provide a more sensitive probe of hydrogen-bonding interactions.

## Supplementary Material





## Notes

Experimental setup for DES preparation, comparison of spectra computed by different electronic structure methods, additional structures and computed spectra for the (2Gly + Cl^–^) complex, computed anharmonic IR spectra, tabulated IR spectral line positions, tabulated relative energies of candidate structures, Gaussian fitting of spectral lines (PDF)
*xyz* coordinates of computed structures, tabulated experimental IR spectra (TXT)
