# Supplementary material for: Halide Coordination Motifs in Deep Eutectic Solvent Building Blocks Characterized by Helium Nanodroplet Infrared Action Spectroscopy
Source: J Phys Chem A. 2026 Aug 11;130(33):6554–64. doi: 10.1021/acs.jpca.6c02885 (PMC13501879; doi:10.1021/acs.jpca.6c02885)
Supplement: Supplementary file 1 [file jp6c02885_si_001.pdf]

Supporting Information

Halide Coordination Motifs in Deep Eutectic Solvent Building Blocks Characterized by Helium Nanodroplet Infrared Action Spectroscopy

Miyuru M. Wellalage,[†,1] Maleesha T. Fernando,[†,1] Katja Ober,[2] América Y. Torres-Boy,[2] Madeline Schultz,[1] Nwanne D. Banor,[1] Emily M. Molino,[1] Jenna E. Lees,[1] Gert von Helden,[2] Daniel A. Thomas[1]*

[1]Department of Chemistry, University of Rhode Island, Kingston, RI 02881 USA

[2]Fritz Haber Institute of the Max Planck Society, 14195 Berlin, Germany

†Authors Contributed Equally

Email: dathomas@uri.edu

Table of Contents

Figure S1: Experimental setup for DES preparation S2

Figure S2: Comparison of the experimental IR action spectrum of Gly + Cl^- with computed IR spectra using different electronic structure methods S2

Figure S3: Comparison of the experimental IR action spectrum of 2Gly + Cl^- with computed IR spectra using different electronic structure methods S3

Figure S4: Experimental and computed IR action spectra of 2Gly + Cl^- S4

Figure S5: Comparison of experimental, harmonic, and anharmonic IR spectra for EG + Cl^- and EG + Br^- S5

Figure S6: Comparison of experimental, harmonic, and anharmonic IR spectra for Gly + Cl^- and 2Gly + Cl^- S6

Figure S7: Comparison of experimental, harmonic, and anharmonic IR spectra for urea + Cl^- and 2urea + Cl^- S7

Figure S8: Comparison of experimental, harmonic, and anharmonic IR spectra for Ch^+ + $2Cl^-$ S8

Table S1: Experimental IR spectral line positions for (EG + Cl^-) and (EG + Br^-) S9

Table S2: Experimental IR spectral line positions for (Gly + Cl^-) and (2Gly + Cl^-) S9

Table S3: Experimental IR spectral line positions for (urea + Cl^-) and (2urea + Cl^-) S9

Table S4: Experimental IR spectral line positions for (Ch^+ + $2Cl^-$) S10

Table S5: Relative energies ($\Delta(E$+ZPE), kJ mol^{-1}) of (2Gly + Cl^-) structures S10

Table S6: Relative energies ($\Delta(E$+ZPE), kJ mol^{-1}) of (2urea + Cl^-) structures S10

Table S7: Relative energies ($\Delta(E$+ZPE), kJ mol^{-1}) of (Ch^+ + $2Cl^-$) structures S10

Table S8: Gaussian spectral line fitting parameters S11

Figure S9: Gaussian fits of spectral lines for selected clusters S12

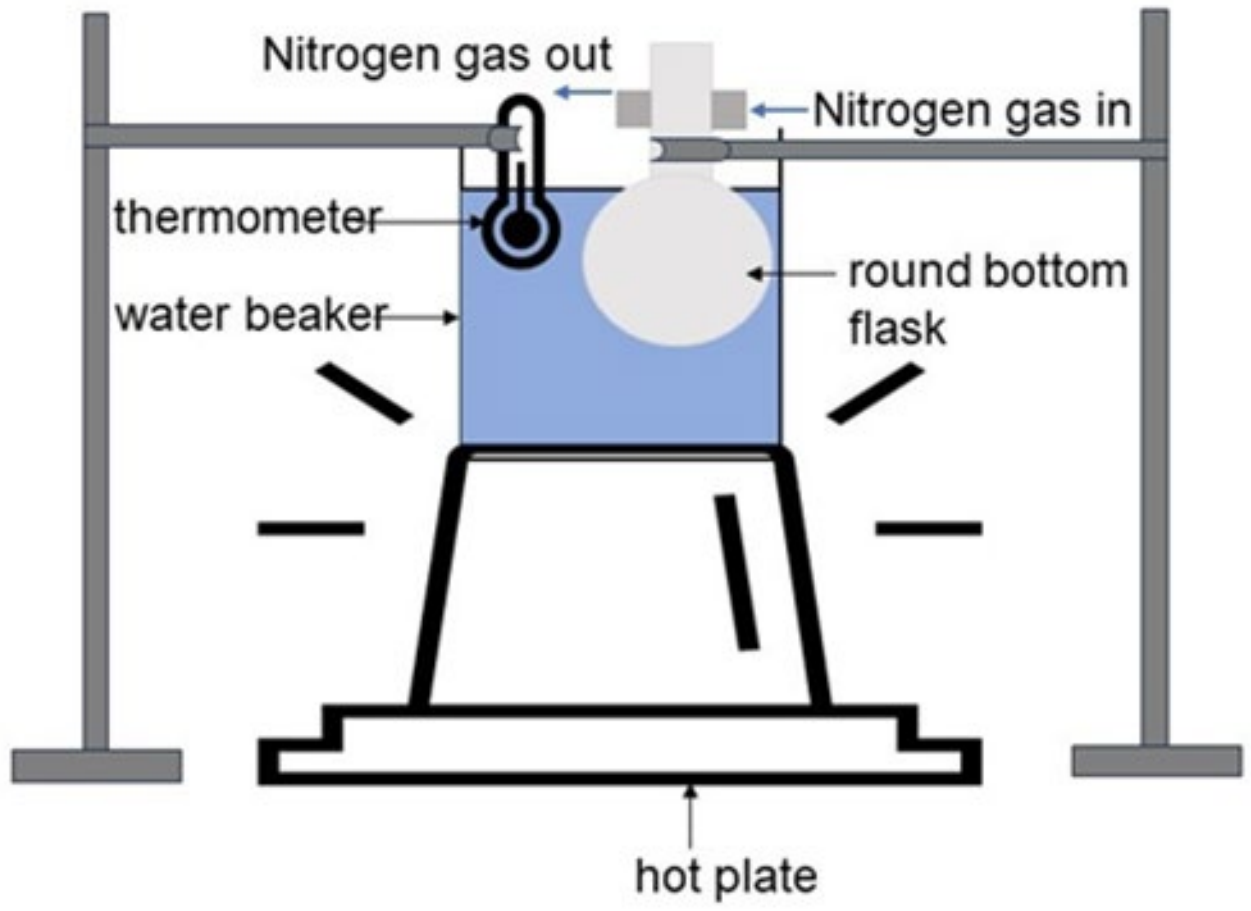

Figure S1. Experimental setup for DES preparation.

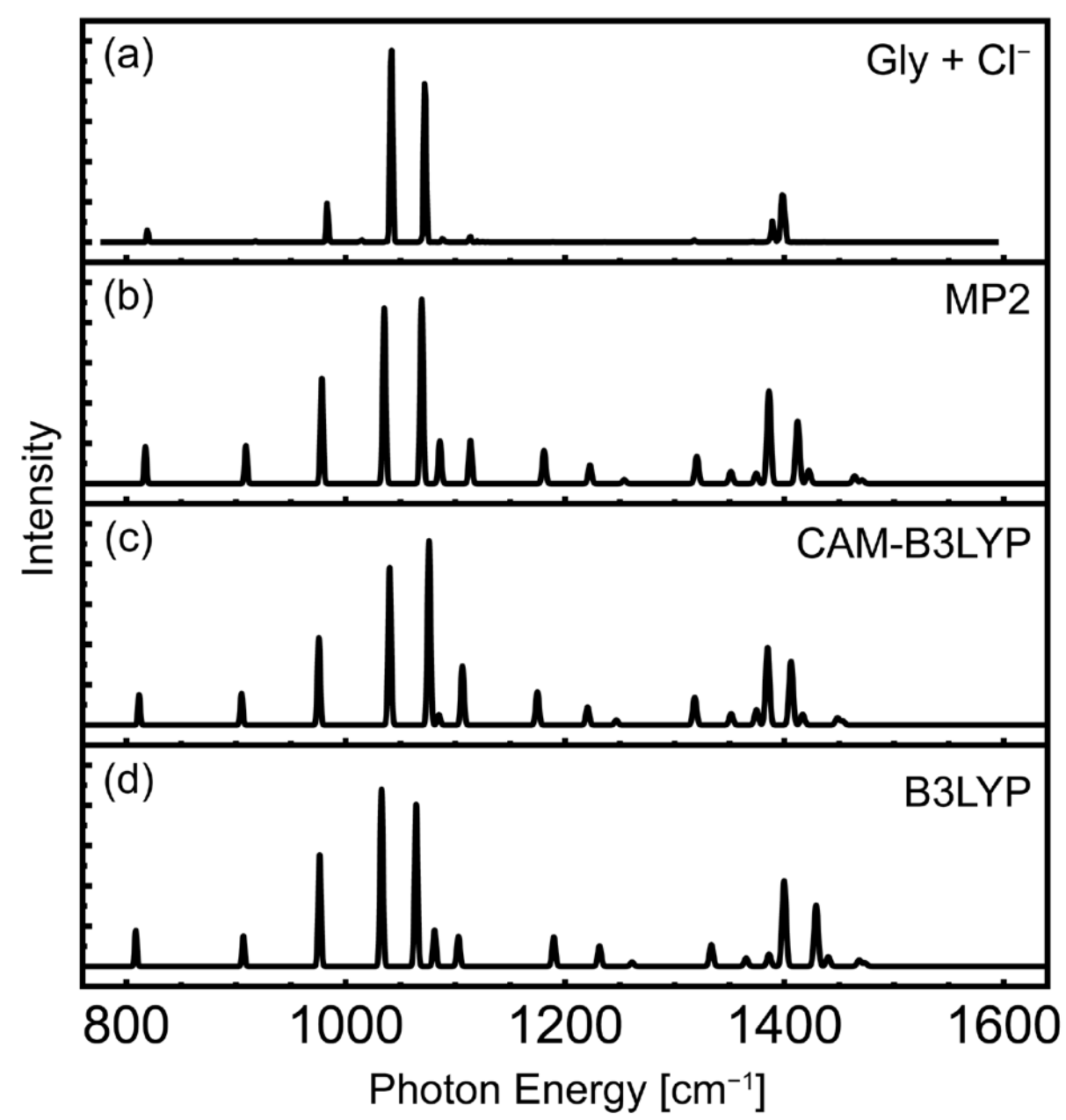

Figure S2. Comparison of the experimental IR action spectrum of Gly + Cl^- (a) with computed IR spectra of structure **3** using the MP2 (b), B3LYP(D3BJ) (c) and CAM-B3LYP(D3BJ) (d) methods. For all computed spectra, the ma-def2-TZVP basis set was used. Scaling factors of 0.97, 0.985, and 0.965 were used for MP2, B3YLP(D3BJ), and CAM-B3LYP(D3BJ), respectively.

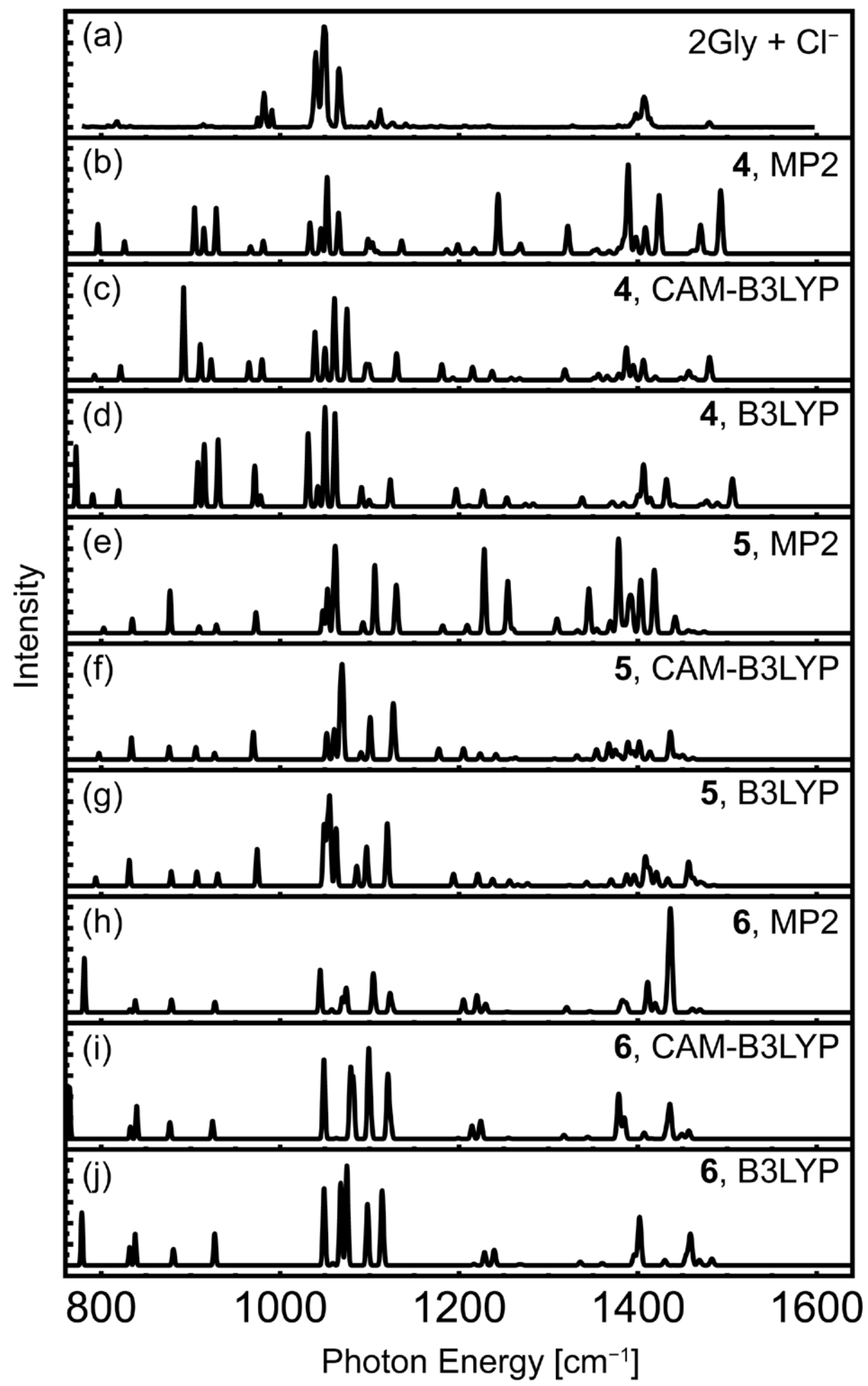

Figure S3. Comparison of the experimental IR action spectrum of 2Gly + Cl^- (a) with computed IR spectra of structures **4**, **5**, and **6** using the MP2 (b, e, h), CAM-B3LYP(D3BJ) (c, f, i) and B3LYP(D3BJ) (d, g, j) methods. For all computed spectra, the ma-def2-TZVP basis set was used. Scaling factors of 0.97, 0.985, and 0.965 were used for MP2, B3YLP(D3BJ), and CAM-B3LYP(D3BJ), respectively.

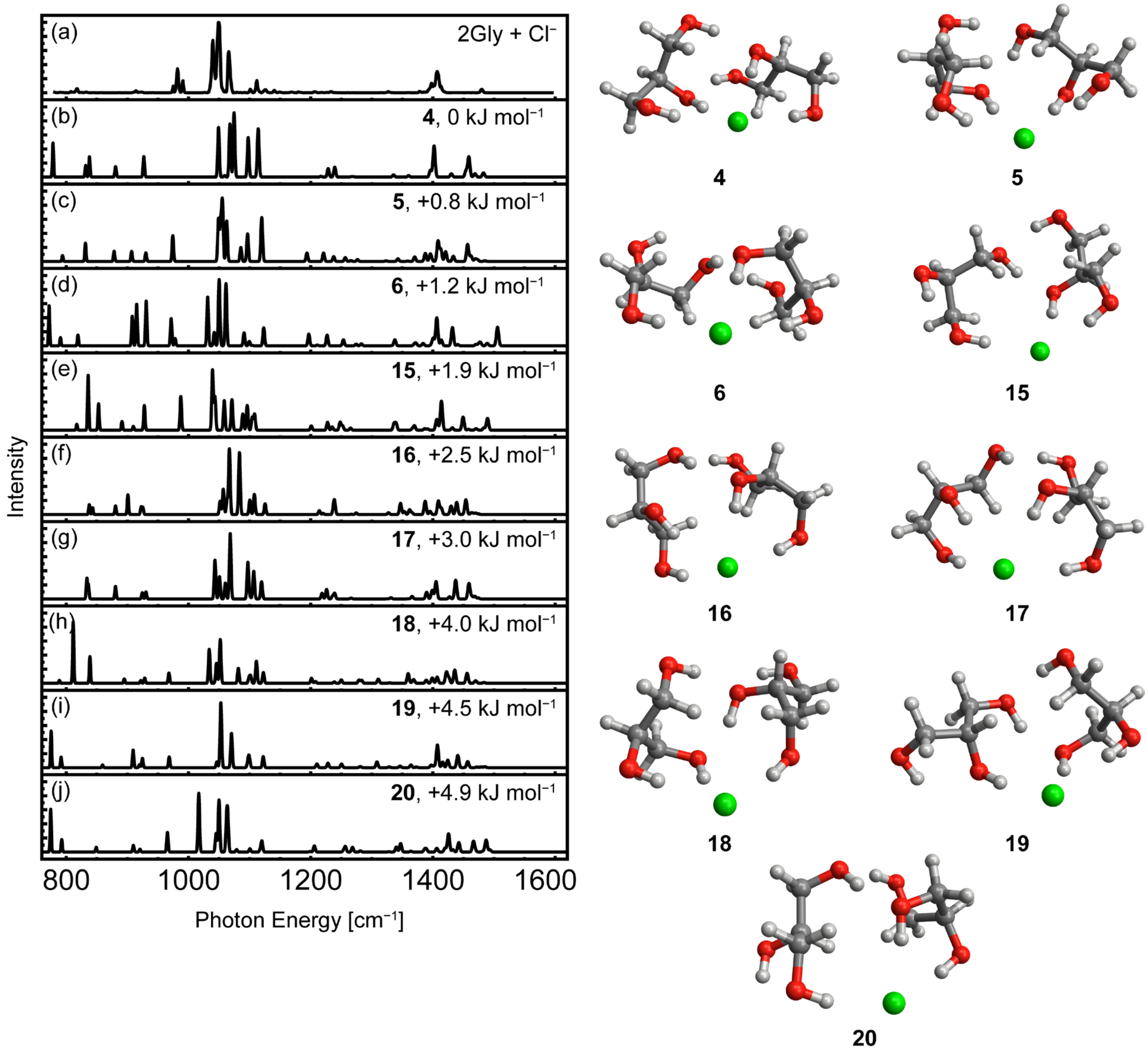

Figure S4. Comparison of the experimental IR action spectrum of 2Gly + Cl^- (a) with computed IR spectra of structures **4–6**, **16–21** (b–j) using the B3LYP(D3BJ)/ma-def2-TZVP method with a scaling factor of 0.985. Corresponding structures are presented to the right of the spectra.

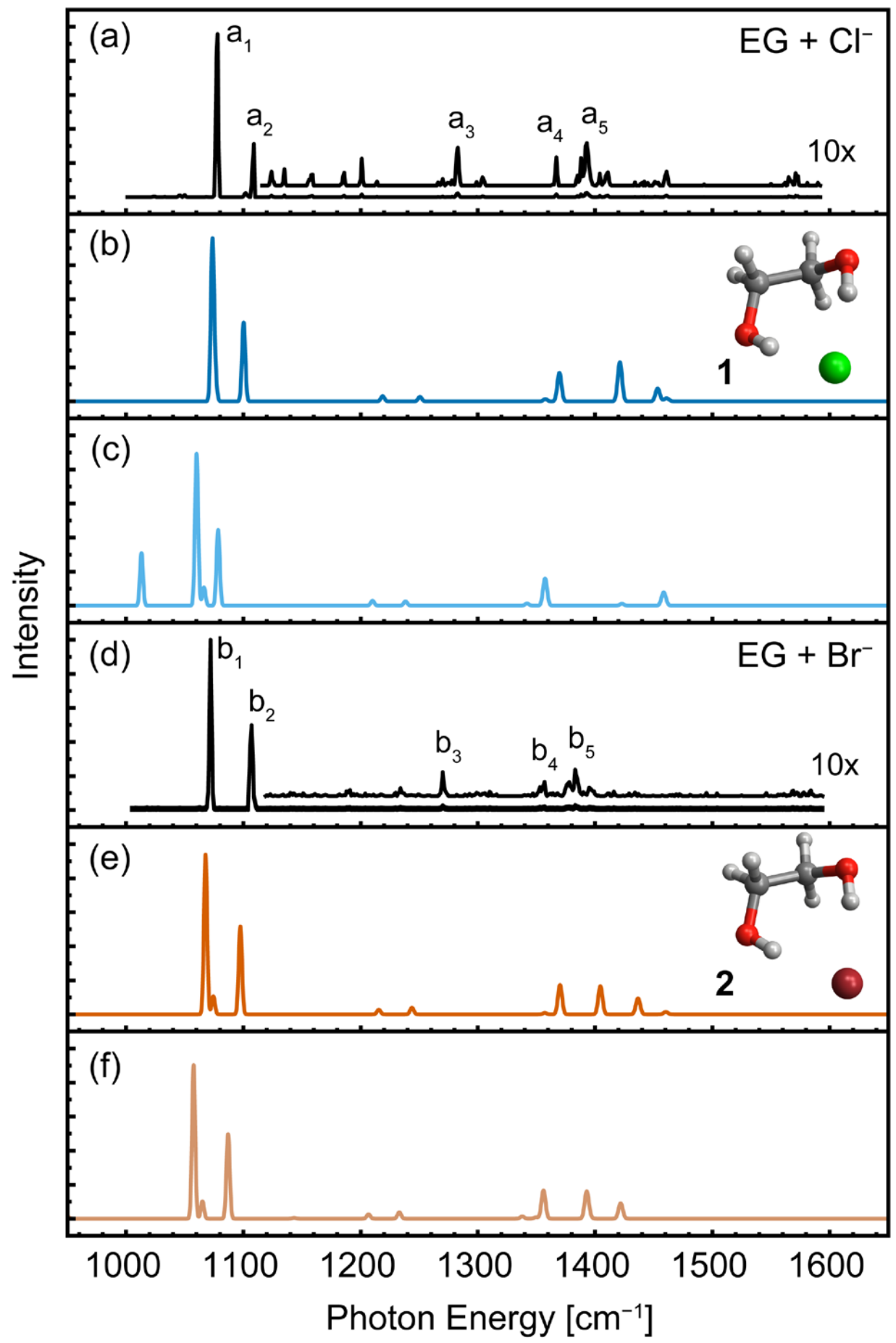

Figure S5. Anharmonic and harmonic IR spectra for EG + Cl^- and EG + Br^-. Shown are the experimental IR action spectrum of EG + Cl^- (a) and computed IR spectra of structure **1** using the harmonic approximation (b, scaling factor of 0.985) and VPT2 anharmonic corrections (c). Also shown are the experimental IR action spectrum of EG + Br^- (d) and computed IR spectra of structure **2** using the harmonic approximation (e, scaling factor of 0.985) and VPT2 anharmonic corrections (f). All theoretical spectra were computed at the B3LYP(D3BJ)/ma-def2-TZVP level of theory.

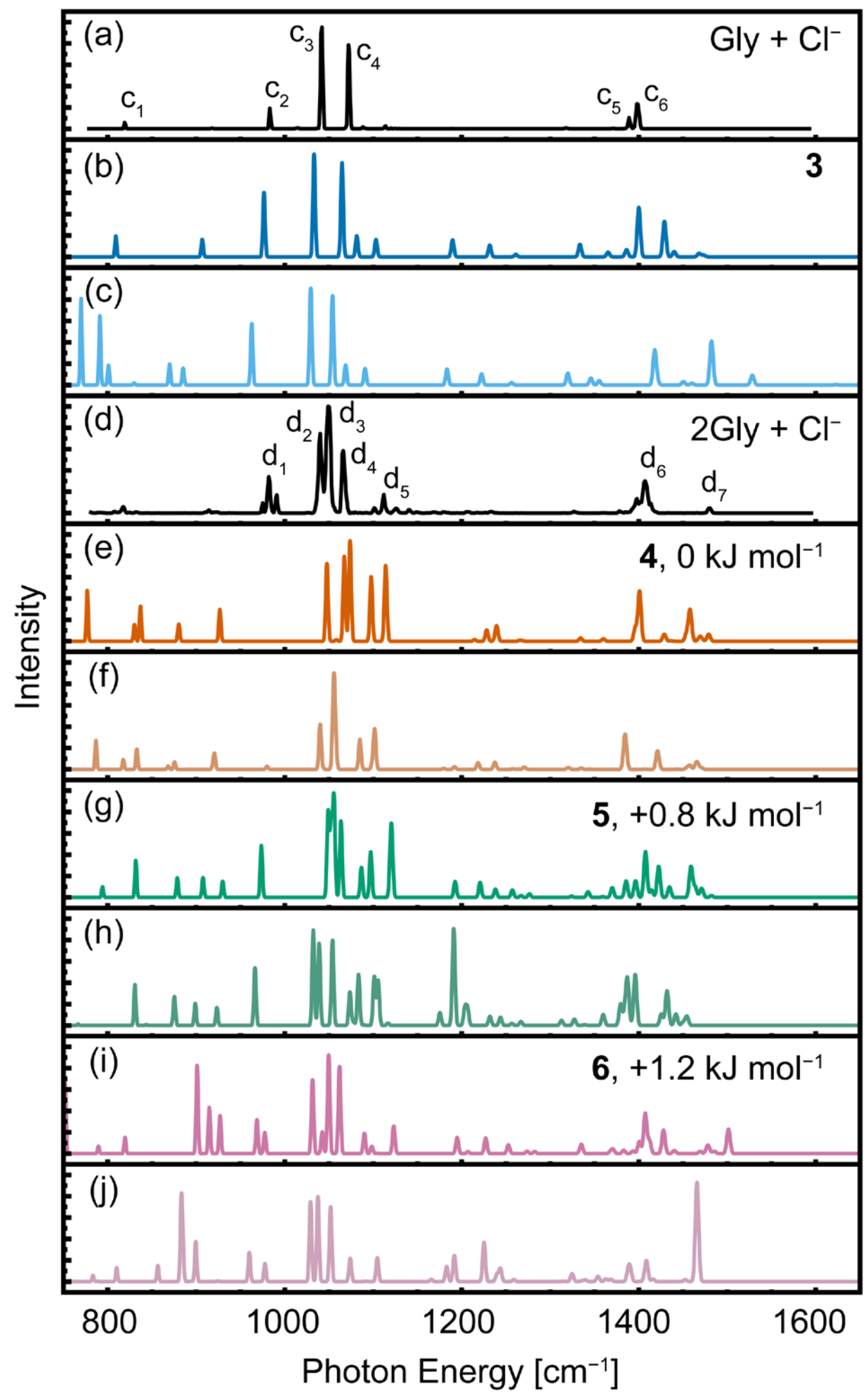

Figure S6. Anharmonic and harmonic IR spectra for Gly + Cl^- and 2Gly + Cl^-. Shown are the experimental IR action spectrum of Gly + Cl^- (a) and computed IR spectra of structure **3** using the harmonic approximation (b, scaling factor of 0.985) and VPT2 anharmonic corrections (c). Also shown are the experimental IR action spectrum of 2Gly + Cl^- (d) and computed IR spectra of structures **4**, **5**, and **6** using the harmonic approximation (e, g, and i, respectively, scaling factor of 0.985) and VPT2 anharmonic corrections (f, h, and j, respectively). All theoretical spectra were computed at the B3LYP(D3BJ)/ma-def2-TZVP level of theory.

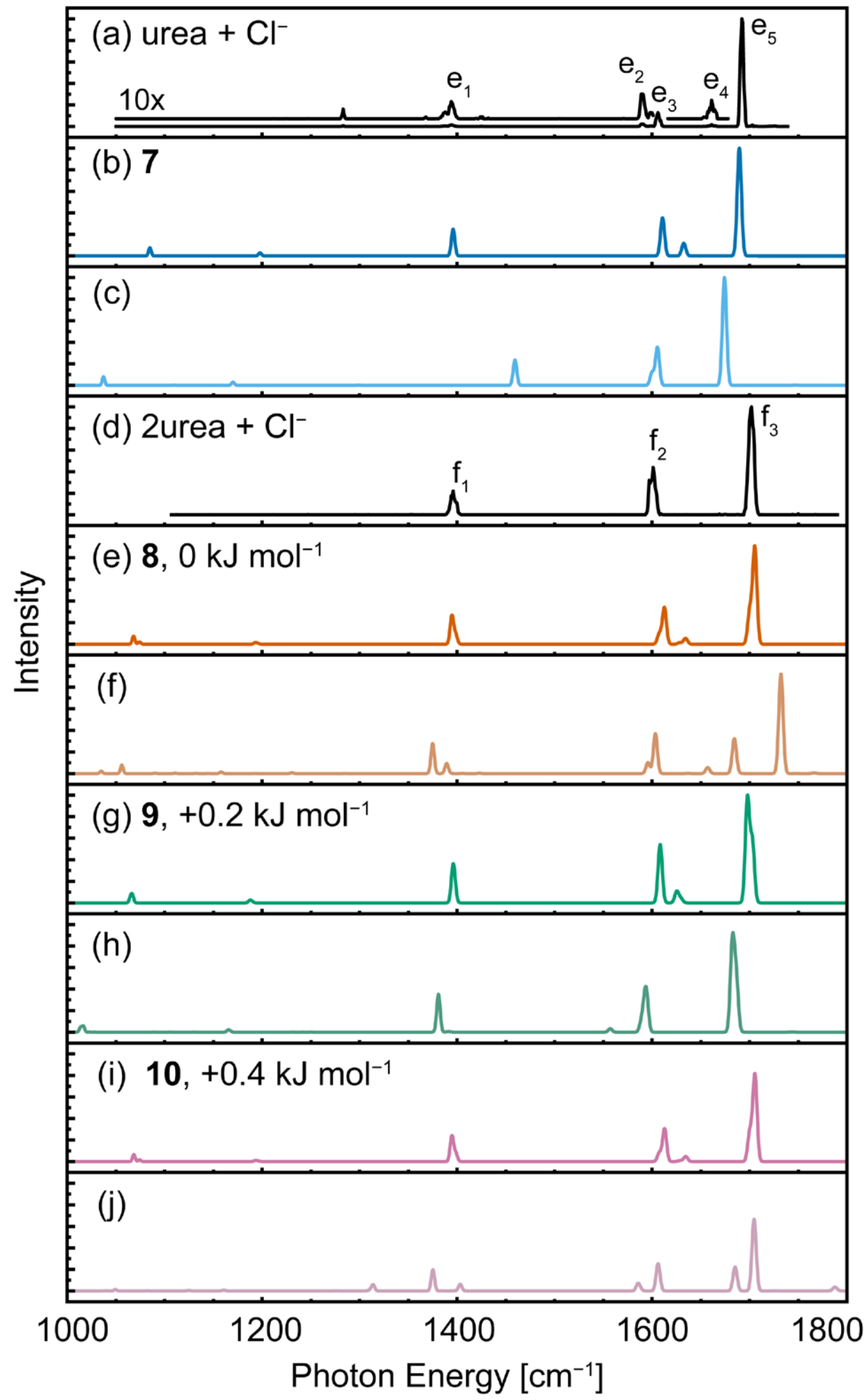

Figure S7. Anharmonic and harmonic IR spectra for urea + Cl^- and 2urea + Cl^-. Shown are the experimental IR action spectrum of urea + Cl^- (a) and computed IR spectra of structure **7** using the harmonic approximation (b, scaling factor of 0.985) and VPT2 anharmonic corrections (c). Also shown are the experimental IR action spectrum of 2urea + Cl^- (d) and computed IR spectra of structures **8**, **9**, and **10** using the harmonic approximation (e, g, and i, respectively, scaling factor of 0.985) and VPT2 anharmonic corrections (f, h, and j, respectively). All theoretical spectra were computed at the B3LYP(D3BJ)/ma-def2-TZVP level of theory.

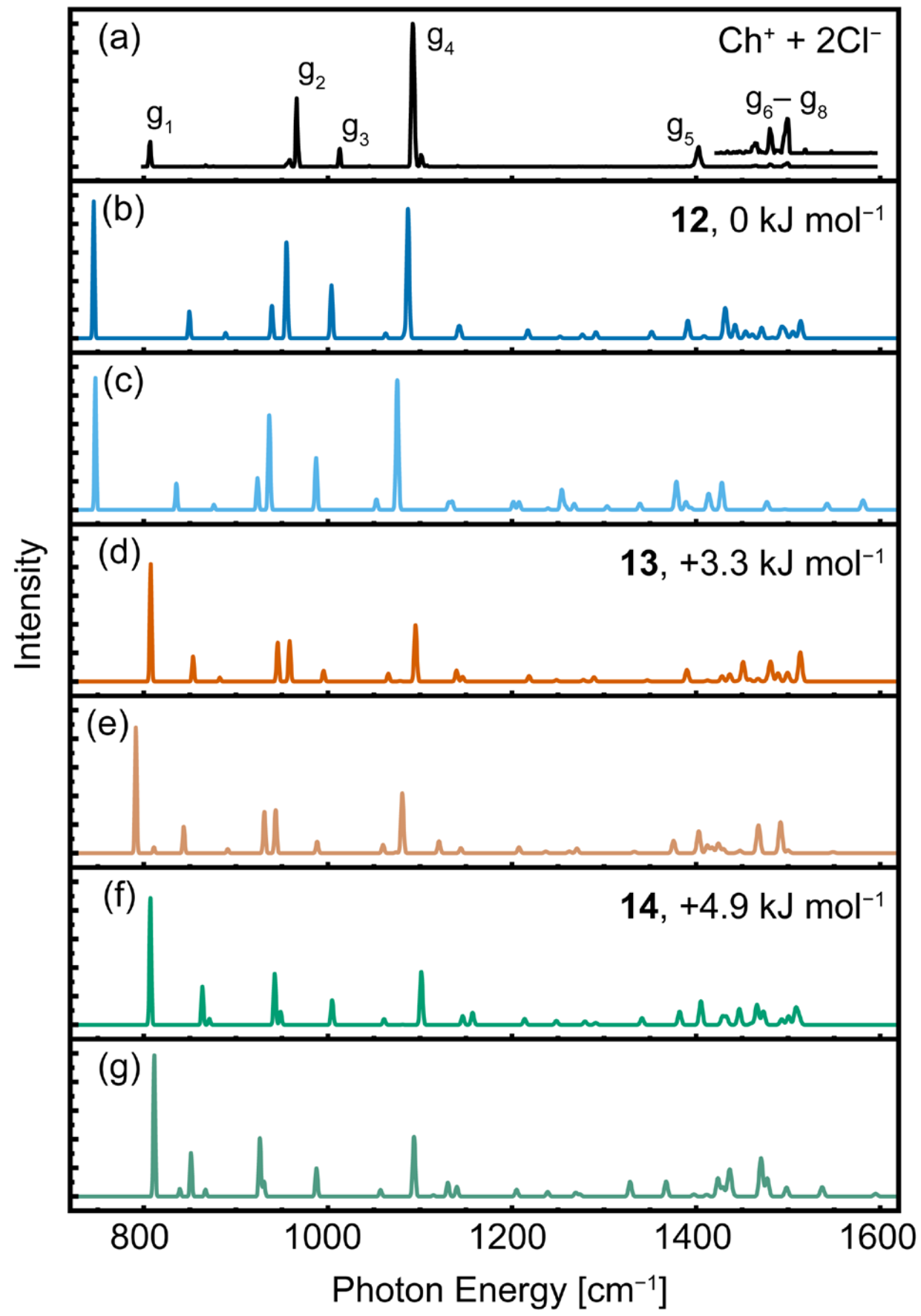

Figure S8. Comparison of the experimental IR action spectrum of Ch^{+} + $2Cl^{-}$ (1) and computed IR spectra of structures **12**, **13**, and **14** using the harmonic approximation (b, d, and f, respectively, scaling factor of 0.985) and VPT2 anharmonic corrections (c, e, and g, respectively). All theoretical spectra were computed at the B3LYP(D3BJ)/ma-def2-TZVP level of theory.

Table S1. Experimental IR spectral line positions for (EG + Cl^-) and (EG + Br^-).

Line	Wavenumber [cm^{-1}]	Line	Wavenumber [cm^{-1}]
a_1	1078	b_1	1072
a_2	1109	b_2	1107
a_3	1283	b_3	1270
a_4	1367	b_4	1357
a_5	1385, 1388, 1393	b_5	1378, 1383

Table S2. Experimental IR spectral line positions for (Gly + Cl^-) and (2Gly + Cl^-).

Line	Wavenumber [cm^{-1}]	Line	Wavenumber [cm^{-1}]
c_1	819	d_1	975, 982, 991
c_2	983	d_2	1040
c_3	1042	d_3	1050
c_4	1072	d_4	1066
c_5	1389	d_5	1102, 1112, 1126, 1141
c_6	1399	d_6	1399, 1407, 1414
		d_7	1480

Table S3. Experimental IR spectral line positions for (urea + Cl^-) and (2urea + Cl^-).

Line	Wavenumber [cm^{-1}]	Line	Wavenumber [cm^{-1}]
e_1	1388, 1394	f_1	1396
e_2	1590	f_2	1601
e_3	1606	f_3	1702
e_4	1661		
e_5	1692		

Table S4. Experimental IR spectral line positions for (Ch^+ + $2Cl^-$).

Line	Wavenumber [cm^{-1}]
g_1	807
g_2	966
g_3	1013
g_4	1092, 1101
g_5	1402
g_6	1464
g_7	1480
g_8	1499

Table S5. Relative energies (Δ(*E*+ZPE), kJ mol^{-1}) of (2Gly + Cl^-) structures **4**–**6**, **15**–**20** (ma-def2-TZVP basis set).

Structure #	B3LYP(D3BJ)	CAM-B3LYP(D3BJ)	MP2
**4**	0.0	+0.2	+0.3
**5**	+0.8	+1.7	+2.0
**6**	+1.2	0.0	0.0
**15**	+1.9	+0.3	+2.3
**16**	+2.5	+1.9	+3.1
**17**	+3.0	+4.0	+5.0
**18**	+4.0	+2.9	+3.4
**19**	+4.5	+2.9	+3.3
**20**	+4.9	+3.2	+3.1

Table S6. Relative energies (Δ(*E*+ZPE), kJ mol^{-1}) of (2urea + Cl^-) structures **8–11** (ma-def2-TZVP basis set).

Structure #	B3LYP(D3BJ)	CAM-B3LYP(D3BJ)	MP2
**8**	0.0	+0.2	0.0
**9**	+0.2	+0.1	+0.1
**10**	+0.4	+0.4	0.0
**11**	+3.9	+3.9	No Minimum

Table S7. Relative energies (Δ(*E*+ZPE), kJ mol^{-1}) of (Ch^+ + $2Cl^-$) structures **12–14** (ma-def2-TZVP basis set).

Structure #	B3LYP(D3BJ)	CAM-B3LYP(D3BJ)	MP2
**12**	0.0	0.0	0.0
**13**	+3.3	+2.8	+1.7
**14**	+4.9	+4.6	+4.3

Table S8. Fitting parameters for Gaussian fitting of spectral lines for (Gly + Cl^-), (2Gly + Cl^-), (urea + Cl^-), and (2urea + Cl^-).

Complex	Fitting Range [cm^{-1}]	Center [cm^{-1}]	Amplitude	Full Width at Half Maximum [cm^{-1}]
(Gly + Cl^-)	975–990	983	15.6	2.1
(Gly + Cl^-)	1036–1078	1042	35.6	2.8
		1072	32.4	2.6
(Gly + Cl^-)	1380–1410	1389	11.1	2.8
		1399	17.4	4.1
(2Gly + Cl^-)	970–995	975	6.0	2.1
		982	11.3	3.7
		991	8.1	2.7
(2Gly + Cl^-)	1030–1075	1040	16.4	4.8
		1049	20.1	5.6
		1066	15.1	4.3
(2Gly + Cl^-)	1385–1425	1398	6.6	8.0
		1407	10.8	5.9
		1414	5.1	4.7
(urea + Cl^-)	1375–1405	1387	1.3	4.5
		1394	2.1	5.5
(urea + Cl^-)	1580–1615	1590	2.7	4.6
		1599	1.4	2.9
		1606	5.9	4.3
(urea + Cl^-)	1685–1700	1692	17.2	3.8
(2urea + Cl^-)	1385–1410	1396	4.9	6.6
(2urea + Cl^-)	1590–1610	1601	3.1	7.2
(2urea + Cl^-)	1690–1715	1702	11.2	6.4

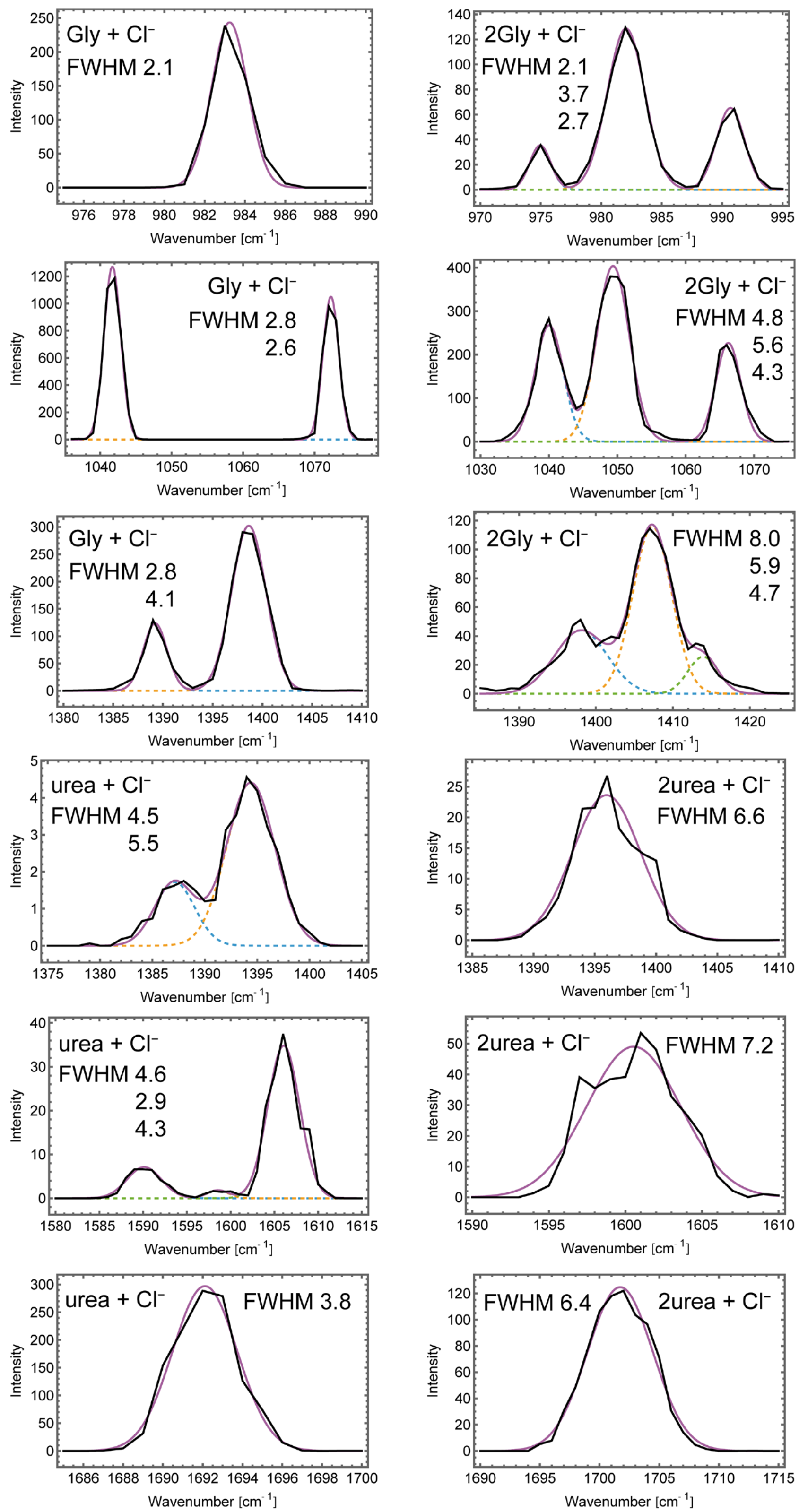

Figure S9. Gaussian fitting (purple) of experimental spectral lines (black) for (Gly + Cl^-), (2Gly + Cl^-), (urea + Cl^-), and (2urea + Cl^-). Full width at half maxima (FWHM) are given.
